# Antimicrobial non-porous surfaces: a comparison of the standards ISO 22196:2011 and the recently published ISO 7581:2023

**DOI:** 10.3389/fmicb.2024.1400265

**Published:** 2024-07-17

**Authors:** Stephanie Maitz, Sabine Poelzl, Daniela Dreisiebner, Eva Zarschenas, Clemens Kittinger

**Affiliations:** Diagnostic and Research Institute for Hygiene, Microbiology and Environmental Medicine, Medical University of Graz, Graz, Austria

**Keywords:** antimicrobial non-porous surface, ISO 22196:2011, ISO 7581:2023, humidity conditions, dry test, *Staphylococcus aureus*, *Escherichia coli*

## Abstract

The application of antimicrobial surfaces requires the proof of their effectivity by *in vitro* methods in laboratories. One of the most well-known test methods is ISO 22196:2011, which represents a simple and inexpensive protocol by applying the bacterial suspension with known volume and concentration covered under a polyethylene film on the surfaces. The incubation is then done under defined humidity conditions for 24 h. Another approach for testing of non-porous surfaces is the newly published ISO 7581:2023. A “dry test” is achieved through spreading and drying 1 μL of a bacterial suspension on the surface. In this study, low alloyed carbon steel, polyethylene terephthalate (PET), and glass specimens were tested uncoated (reference) and coated with zinc according to both ISOs to compare and to evaluate the advantages and disadvantages of each one of them. Although ISO 7581:2023 allows a more realistic test environment than ISO 22196:2011, the reproducibility of the results is not given due to the low application volume. In addition, not all bacterial strains are equally suitable for this testing type. Individual adaptations to the protocols, including incubation conditions (time, temperature, or relative humidity), testing strains and volume, seem necessary to generate conditions that simulate the final application. Nevertheless, both ISOs, if used correctly, provide a good basis for estimating the antimicrobial efficacy of non-porous surfaces.

## 1 Introduction

Prevention of microbial contamination and awareness of antimicrobial surfaces have received more attention following the SARS-CoV-2 pandemic. However, for bacterial diseases to occur, the pathogen must first be transmitted to the potential host. In this respect, most microorganisms are moved passively in the environment, including aerosols (Mirhoseini et al., 2015) or contact between an uncontaminated and contaminated object (Stephens et al., 2019). Likewise, microorganisms are not only transferred, some maintain their pathogenic potential outside of their host (Pommepuy et al., 1996) and even survive for days or weeks on non-porous surfaces such as glass, plastic, or stainless steel (Neely, 2000; Heller and Edelblute, 2018; Katzenberger et al., 2021). Especially solid materials are popular in clinical facilities, food service industries, and other sterile facilities and systems (e.g., ventilating systems, laboratories, etc.), as they represent one key to control hygiene and product quality (Detry et al., 2010). However, the transfer of potentially harmful microorganisms from these non-porous surfaces to a biological contact surface (e.g.: humans), by touch, is not unknown. The surfaces provide the potential reservoir to cause infection or cross-infection between, e.g., humans (Suwantarat et al., 2017; Kraay et al., 2018; Weber et al., 2020).

In order to reduce bacterial transfer and control disease outbreaks without compromising the availability of facilities and systems, research on antimicrobial materials (AMMs) has been done and is ongoing. Different mechanisms of action of these materials are already on the market and can be divided into three groups: active substance release AMMs systems, potentiated surface-based AMMs (include biocides, metals, peptides, or amines on their surfaces) and finally non-adhesive AMMs (Campoccia et al., 2013; Sjollema et al., 2018; Cunliffe et al., 2021). Due to the different materials and additives, as well as the different environmental conditions in the respective application areas, it should be precisely evaluated and determined by means of test procedures which AMMs can be used for which intended application area, in order to achieve an antimicrobial effect (Cunliffe et al., 2021).

However, the standardized *in vitro* test methods to verify antimicrobial activity are just as diverse as antimicrobial materials and additives available on the market. These test methods can be divided into five categories according to their mechanism of action, they are intended to evaluate, including high surface-to-volume ratio tests, agar inhibition zone tests, suspension tests, adhesion tests, and biofilm tests (Sjollema et al., 2018). Selecting the appropriate test methods from this range is anything but trivial. Attention should be made at this point, as antimicrobial surface activity has been reported to vary depending on the testing protocol (Campos et al., 2016). Thus, again wariness is required which method is used to test the antimicrobial activity of AMMs and also seems appropriate for the application of the respective AMMs (Cunliffe et al., 2021). One of the most well-known test methods in the industry is the International Standards Organization (ISO) 22196:2011 (International Organization for Standardization, 2011) and the equivalent Japanese version Japanese Industrial Standard (JIS) Z 2801:2010 (Japanese Industrial Standards Association, 2010), which represent the first category of AMMs test methods. They represent a simple and inexpensive protocol and are therefore widely applied to test antimicrobial activity on non-porous surfaces. With ISO 22196:2011, a precisely defined surface of the specimens to be tested is inoculated with a known volume and concentration of a bacterial suspension and covered with a polyethylene film. The inoculated specimens are then incubated for 24 h at 35°C ± 1°C and a relative humidity (RH) of not less than 90%. Afterward, the bacterial suspension is rinsed off the surface of the specimens, diluted and plated on agar plates to determine the number of viable bacteria by counting the plates, again to determine the antimicrobial activity of the specimens. Even though the protocol seems reliable and easy to follow, there are some consistently discussed critical points of ISO 22196:2011. Wiegand et al. (2018) established already a round robin test on this standard protocol and they highlighted four critical factors, which are influencing the outcome of the antibacterial testing: (1) incubation time, (2) bacteria starting concentration, (3) physiological state of bacteria (stationary or exponential phase of growth), and (4) nutrient concentration. Moreover, the specified test conditions with 90% relative humidity, leaving the polyethylene film covered specimens wet throughout the test period, is known to artificially promote an antimicrobial activity. For instance, the antimicrobial activity of metallic silver, can be increased by high humidity over a long incubation period by forming silver ions (Noyce et al., 2006; Michels et al., 2009). In addition, the antimicrobial agents contained in the AMMs dissolve in liquid (Bäumler et al., 2022) and can thus interact with the bacteria more easily and exert their effect more effectively. These moisturized conditions and the fully contact of the microorganisms with the antimicrobial surface by the polyethylene film might not mimic realistic environmental conditions in which the product is to be applied. Therefore, reproducible conditions that simulate the end-use environment of the material are recommended (Cunliffe et al., 2021). As described in a recently publised review of ISO 22196:2011, the limitations in representing real-world conditions of this standardized method require further research and modification (Bento de Carvalho et al., 2024). An important point in adapting the test method is testing antimicrobial activity after the bacterial suspension has dried on the specimens. For this purpose, knowledge of the duration of the drying time of the inoculum is important in order to accurately assess the efficacy of the surface (Rai et al., 2009; Cunliffe et al., 2021). To overcome these limitations, a new test method has recently been published, ISO 7581:2023 (International Organization for Standardization, 2023), to test non-porous AMMs in a dry environment. This time, a precisely defined very small volume and concentration of a bacterial suspension is distributed on the surface of the tested specimens. The inoculum is dried under a laminar flow hood for defined time. Then, the inoculated specimens are incubated for 1–2 h at 20°C ± 1°C and a RH of below 90% (30–65%). With these changes in the test method, the artificial antimicrobial activity derived from a wet microbial inoculum and environment as in ISO 22196:2011 is reduced. Afterward, the protocol is almost similar to ISO 22196:2011 for determining the recovery of viable bacterial and to analyze the antimicrobial effect. However, also this protocol has its shortcomings when it comes to ensuring the reproducibility of the amount of dry bacterial suspension (Cunliffe et al., 2021).

Therefore, the aim of this article is to review the two ISO protocols ISO 22196:2011 and ISO 7581:2023 and their different approach to test non-porous surfaces, to compare them and to evaluate the advantages and disadvantages.

## 2 Materials and methods

### 2.1 Specimen selection and pre-treating

Three non-porous samples were analyzed in this study, including low alloyed carbon steel (voestalpine Stahl GmbH, Linz, Austria), polyethylene terephthalate (PET) film (INOCON Technologie GmbH, Attnang-Puchheim, Austria), and glass (slide 50 mm × 50 mm × 1.55 mm, Cloeren Technology GmbH, Wegberg, Germany). All samples had the same size of 50 mm × 50 mm and were tested both uncoated as reference and coated with zinc (Supplementary Figure 1). The low alloyed carbon steel and glass surfaces were coated by voestalpine Stahl GmbH (Linz, Austria) using physical vapor deposition (PVD) technology with a target layer thickness of 2 μm zinc. The PET film was coated using atmospheric pressure plasma deposition (APPD) with an INOCON InoCoat3 Plasma Jet (INOCON Technologie GmbH, Attnang-Puchheim, Austria) with an approximately 36% loading of the surfaces with zinc particles. The samples were individually packed in sealed sterile plastic bags to ensure sterility during transport to the microbiological laboratory. There the samples were stored at room temperature. Prior to testing, the samples were sterilized with 70% ethanol (Merck KGaA, Darmstadt, Germany).

### 2.2 Testing of antibacterial activity

#### 2.2.1 ISO 22196:2011 measurement of antibacterial activity on plastics and other non-porous surfaces

In order to enable a better comparison between the two ISO protocols, small adjustments were made to the test setup of ISO 22196:2011. ISO 22196:2011 and ISO 7581:2023 use different strains for testing of the antimicrobial activity, in order to be able to compare the protocols, all four strains were tested in each protocol: *Staphylococcus aureus* (*S. aureus*) DSM 346, *S. aureus* DSM 799, *Escherichia coli* (*E. coli*) DSM 1576, and *E. coli* DSM 682 (Leibniz Institute DSMZ—German Collection of Microorganisms and Cell Cultures GmbH, Braunschweig-Sued, Germany). They were cultivated overnight (16–20 h) on Columbia Blood Agar plates (Becton Dickinson GmbH, Heidelberg, Germany) at 36°C ± 2°C The cell material was inoculated in a 0.2% Tryptone Soy Broth (TSB, Oxoid Limited, Hampshire, United Kingdom) diluted in distilled water. A VITEK® DensiCHEK device (bioMerièux Austria GmbH, Vienna, Austria) was used to obtain a bacterial solution with 10^8^ colony forming units (CFU)/mL. In order to obtain an initial concentration of 2.5 × 10^5^–10 × 10^5^ CFU/mL, the bacterial suspension was diluted accordingly in 0.2% Tryptone Soy Broth. To quantify the bacterial test suspension concentration, the adjusted bacterial suspensions were serial diluted with 1x Phosphate Buffered Saline (PBS, Carl Roth GmbH + Co Kg, Karlsruhe, Germany) to 10^−4^ and 10^−5^. A duplicate of 500 μL from each dilution was plated onto Tryptic Soy Agar (TSA, VWR International GmbH, Darmstadt, Germany) plates by spread plate technique. After incubation for 24 h at 36°C ± 2°C, one dilution containing 30–300 CFU on the plates was selected and counted. The weighted mean bacterial concentration was calculated in CFU/cm^2^ with the following formula:


$$X=\left(100*C*2*D\right)/A$$

*X* is an initial suspension concentration (applied load) in CFU/cm^2^.

*C* is average plate count for the duplicate plates (*2 was added for the conversion of 500 μL plated to 1 mL).
*D* is dilution factor for the plates counted.
*A* is the surface area, in mm^2^, of the cover film (1,600 mm^2^).

Each specimen was placed into a separate sterile petri dish (90 mm × 16.2 mm, Fisher Scientific, Schwerte, Germany) with the test surface facing upwards. Then, 200 μL bacterial suspensions with an expected bacterial concentration of 2.5 × 10^5^–10 × 10^5^ CFU/mL were pipetted on the sterile surfaces of the samples. If the test inoculum was not used immediately, it was chilled on ice and used within 2 h after preparation. The bacterial suspensions applied on the surface were covered under a sterile 40 mm × 40 mm polyethylene film (VWR International GmbH, Darmstadt, Germany), which was ligthly pressed down to distribute the bacterial suspension evenly under the polyethylene film. The petri dishes were then closed and incubated in a climate chamber at 35°C ± 1°C with a relative humidity (RH) of above 90% for 1 and 24 h. Both temperature and RH were constantly digitally monitored. After exposure, the bacterial suspension on each specimen was rinsed four times with 10 mL of neutralizer called Soybean Casein Digest broth with Lecithin and Polyoxyethylene sorbitan monooleate (SCDLP broth) containing TSB (Oxoid Limited, Hampshire, United Kingdom), lecithin (Carl Roth GmbH + Co Kg, Karlsruhe, Germany) and Tween®80 (Amresco Inc., Solon, Ohio, United States) in order to rescue surviving bacteria. In addition to the protocol of ISO 22196:2011 and for better comparability with ISO 7581:2023, the specimens were shaken in the neutralization medium for 3 min with 200 rpm on a Battery Shaker KM 2 Akku (Edmund Bühler GmbH, Bodelshausen, Germany). The dilution series was performed according to protocol in 1x PBS (Carl Roth GmbH + Co Kg, Karlsruhe, Germany). 500 μL of appropriate dilutions were plated directly on TSA (VWR International GmbH, Darmstadt, Germany) plates in duplicates by spread plate technique. The plates were incubated for 24 h at 36°C ± 2°C. After incubation either a dilution with 30–300 CFU was selected or if less than 30 CFU were present these were also counted and the amount recorded. If colonies could not be counted on any of the agar plates, the detection limit was set at 6.25 × 10^0^ CFU/cm^2^, as ISO 22196:2011 requires “< V” to be reported in this case. If less than 6.25 × 10^0^ CFU/cm^2^ were calculated for a replicate, the same limit of detection was set.

The applied bacterial load is defined as the actual number of bacterial cells applied to the specimens in the experiment. Time point 0 h is defined as the test point where the bacterial suspension is harvested immediately after pipetting under the foil to ensure initial concentration of each sample and to validate loss due to manipulation. For each incubation time, triplicates (*n* = 3) were used to calculate mean and standard deviation for the antibacterial activity of the specimens.

For each test sample, the recovered number of viable bacteria in CFU/cm^2^ was calculated using the following formula:


$$N=\left(100*C*2*V*D\right)/A$$

N is the number of viable bacteria recovered per cm^2^ per test specimen [CFU/cm^2^].

C is an average plate count for the duplicate plates (*2 was added for the conversion of 500 μL plated to 1 mL).
V is volume, in mL, of SCDLP added to the specimen (10 mL).
A is the surface area, in mm^2^, of the cover film (1,600 mm^2^).

Calculation of the reduction was done with following formula:


$$R=\left(U_{t}-U_{0}\right)-\left(A_{t}-U_{0}\right)=U_{t}-A_{t}$$

*R* is the antibacterial activity.

U_0_ is the average of the common logarithm of the number of viable bacteria, in cells/cm^2^, recovered from the untreated test specimens immediately after inoculation.
U_t_ is the average of the common logarithm of the number of viable bacteria, in cells/cm^2^, recovered from the untreated test specimens after 1/24 h.
A_t_ is the average of the common logarithm of the number of viable bacteria, in cells/cm^2^, recovered from the treated test specimens after 1/24 h.

The verification of the methodology was calculated according to the guideline through the 0 h triplicates with the following formula:


$$\frac{\left(L_{\text{max}}-L_{\text{min}}\right)}{L_{\mathrm{mean}}}\leq 0.2$$

L_max_ is 10 logarithm of the maximum number of viable bacteria found on a specimen.

L_min_ is 10 logarithm of the minimum number of viable bacteria found on a specimen.
L_mean_ is 10 logarithm of the mean number of viable bacteria found on the specimens.

A value ≤0.2 indicated a valid test result.

Further conditions for a valid test are described for the reference samples as follows:

The number of viable bacteria recovered at time point 0 h should be within the range of 6.2 × 10^3^ to 2.5 × 10^4^ CFU/cm^2^.
After 24 h, the results should not be less than 6.2 × 10^1^ CFU/cm^2^.

#### 2.2.2 ISO 7581:2023 evaluation of bactericidal activity of a non-porous antimicrobial surface used in a dry environment

In order to enable a better comparison between the two ISO protocols, a few adjustments were made to the test setup of ISO 7581:2023. Information on the used strains was already given in section 2.2.1. The cell material was inoculated in a 0.85% Tryptone Sodium Chloride (Tryptone NaCl, Carl Roth GmbH + Co Kg, Karlsruhe, Germany) solution diluted in distilled water. Again a VITEK® DensiCHEK device (bioMerièux Austria GmbH, Vienna, Austria) was used to obtain a bacterial solution with 1.5 × 10^8^–5 × 10^8^ CFU/mL. To quantify the concentration of the suspension, the adjusted bacterial liquid cultures were diluted with 0.85% Trypton NaCl (Carl Roth GmbH + Co Kg, Karlsruhe, Germany) to 10^−6^ and 10^−7^. A duplicate of 500 μL was taken from each dilution and plated onto TSA (VWR International GmbH, Darmstadt, Germany) plates by spread plate technique. The plates were incubated for 24 ± 2 h at 35°C to 38°C ± 1°C. All plates containing 15–300 CFU ± 10% CFU after incubation were counted. The weighted mean bacterial concentration was calculated in CFU using the following formula:


$$X=\frac{\left(c*2\right)}{\left(n_{1}+0.1*n_{2}\right)}*d$$

*X* is initial suspension concentration (applied load) in CFU.

*c* is the sum total of the values of viable cultivable bacteria (V_c_) considered (*2 was added for the conversion of 500 μL plated to 1 mL).
*n*_1_ is the number of values of V_c_ considered in the lowest dilution.
*n*_2_ is the number of values of V_c_ considered in the highest dilution.
*d* is the dilution level corresponding to the lowest dilution.

Each specimen was placed into a separate sterile petri dish (90 mm × 16.2 mm, Fisher Scientific, Schwerte, Germany) with the test surface facing upwards. Then, 1 μL of the bacterial suspensions with an expected bacterial concentration of 1.5 × 10^8^–5 × 10^8^ CFU/mL was pipetted on the sterile surfaces of the sample. The inoculum was spread on the individual samples using the pipette tip. If the test inoculum was not used immediately, it was chilled on ice and used within 2 h after preparation. The bacterial suspension was then dried in the laminar flow (Kojair Tech Oy, Mänttä-Vilppula, Finland) for 3–10 min. The contact time was recorded after drying, the petri dishes were closed and incubated in a climate chamber at 20°C ± 1°C with a RH between 30 and 65% for 1 or 24 h. Both temperature and RH were constantly digitally monitored. After exposure, each sample was transferred into a plastic container with 10 mL of recovery liquid SCDLP broth prepared as described in ISO 22196:2011 and 12–14 g glass beads (2.85–3.3 mm, Carl Roth GmbH + Co Kg, Karlsruhe, Germany) with the test surface facing downwards to rescue surviving bacteria. In addition, the samples were shaken in the recovery liquid for 3 min with 200 rpm on a Battery Shaker KM 2 Akku (Edmund Bühler GmbH, Bodelshausen, Germany). The dilution series were performed according to the protocol in 0.85% Tryptone NaCl (Carl Roth GmbH + Co Kg, Karlsruhe, Germany) solution. Finally, 500 μL of appropriate dilutions were plated directly on Tryptic Soy Agar (TSA, VWR International GmbH, Darmstadt, Germany) plates in duplicates by spread plate technique. All plates with 15–300 ± 10% CFU after incubation for 24 ± 2 h at 35°C to 38°C ± 1°C were counted.

The experimental validation (V_n_) was done for each strain and sample tested. 1 μL of 0.85% Tryptone NaCl (Carl Roth GmbH + Co Kg, Karlsruhe, Germany) was pipetted on the different sample surfaces. After drying, the samples were transferred in a plastic container containing 10 mL SCDLP broth and 12–14 g glass beads. Then, 1 μL of the test suspension was added. The further procedure was the same as described above.

The applied load is defined as the actual number of bacterial cells applied to the sample in the experiment. The bacterial counts corresponding to time 0 h belong to the samples where the bacterial suspension was preferably dried for 3–10 min under the laminar flow and then harvested to ensure the initial applied concentration to validate any loss due to manipulation or the drying process. For each incubation time (C_0h_ and C_xh_ for reference, T_0h_ and T_xh_ for zinc-coated specimens) all experiments were carried out in triplicates (*n* = 3) and used to calculate mean and standard deviation for the antibacterial activity. Bacterial load is expressed in CFU. The determination of the number of viable bacteria was calculated using following formula:


$$C_{0h},C_{xh},T_{0h},T_{xh},V_{n}=\frac{\left(c*2\right)}{\left(n_{1}+0.1*n_{2}\right)}*10*d$$

*c* is the sum total of the values of viable cultivable bacteria (V_c_) considered in CFU (*2 was added for the conversion of 500 μL plated to 1 mL).

*n*_1_ is the number of values of Vc considered in the lowest dilution.
*n*_2_ is the number of values of Vc considered in the highest dilution.
10 is the dilution factor corresponding to the recovery in 10 mL of recovery liquid.
*d* is the dilution level corresponding to the lowest dilution.

For two scenarios, the filtration technique and sample surface transfer to a TSA plate were used for the calculation of the result. Once, if all the numbers of CFU on the agar plates were < 14 CFU. Then, the counts by filtration (F) and by pour plate of the surface (S) in the agar, were considered in following formula:


$$\frac{F*10}{Fv}+S$$

*F* is a total number of CFU counts by filtration.

Fv denotes volume filtrated.
*S* is total number of CFU by pour plate of the surface in the agar.

Second, if the number of CFU of 10^0^ and of the count by filtration are >14 following formula was used:


$$\frac{\left(\frac{F*10}{Fv}+\frac{c}{n_{0}}\right)}{1.1}+S$$

*F* is total number of CFU counts by filtration.

*F*v denotes volume filtrated.
*c* is total number of CFU on the plates of 10^0^.
*n*_0_ is the number of values of c taken into account.
*S* is total number of CFU by pour plate of the surface in the agar.

For the filtration the remaining SCDLP broth (approximately 7 mL, after preparation of the dilution series) was filtered with a sterile filtration device using an EZ-Stream™ vacuum pump coupled to EZ-fit 3-way manifold (Merck KGaA, Darmstadt, Germany). The used 0.45 μm EZ-Pak membrane filter (Merck KGaA, Darmstadt, Germany) was then transferred to a TSA plate and incubated at 35°C to 38°C ± 1°C for 24–48 h (Supplementary Figure 2). In addition, the test surface was transferred on a TSA plate and covered with 10 mL of liquid TSA medium (Supplementary Figure 3). When no CFU were countable on each dish, including counts by filtration and counts from agar-embedded surface, the final value of C_xh_/T_xh_ was set to 0 CFU.

Calculation of the reduction was done with following formula:


$$R=C_{xh}-T_{xh}$$

R is reduction expressed as a base ten logarithm.

Following verification steps are used for ISO 7581:2023:

X (the initial suspension) shall be between 1.5 × 10^8^ and 5 × 10^8^ CFU.
*N* (number of theoretical CFUs deposited on the surface) shall be between 1.5 × 10^5^ and 5 × 10^5^ CFU.
*V*_n_ shall differ from N by <2 log – the difference between the surfaces shall not be >0.3 log.

C_0_ triplicates were used to verify the methodology according to the guideline through the following formula:


$$\frac{\left(C_{0h,\max}-C_{0h,\min}\right)}{\left(C_{0h,\mathrm{mean}}\right)}\leq 0.3$$

C_0h, max_ is 10 logarithm of the maximum number of viable bacteria found on a specimen.

C_0h, min_ is 10 logarithm of the minimum number of viable bacteria found on a specimen.
C_0h, mean_ is 10 logarithm of the mean number of viable bacteria found on the specimens.

A value ≤0.3 indicated a valid test result.

Further calculation for the reference surfaces have been done. The mean of C_0h_ and C_xh,_ were each subtracted from the corresponding standard deviation (SD). The value of the mean log minus SD shall be ≥3.

S should be less than 100 CFU for active (coated) surfaces.

Check of the counts obtained by the weighted mean: the quotient is neither less than 5 nor more than 15.

#### 2.2.3 Comparing both protocols

**Figure 1:**
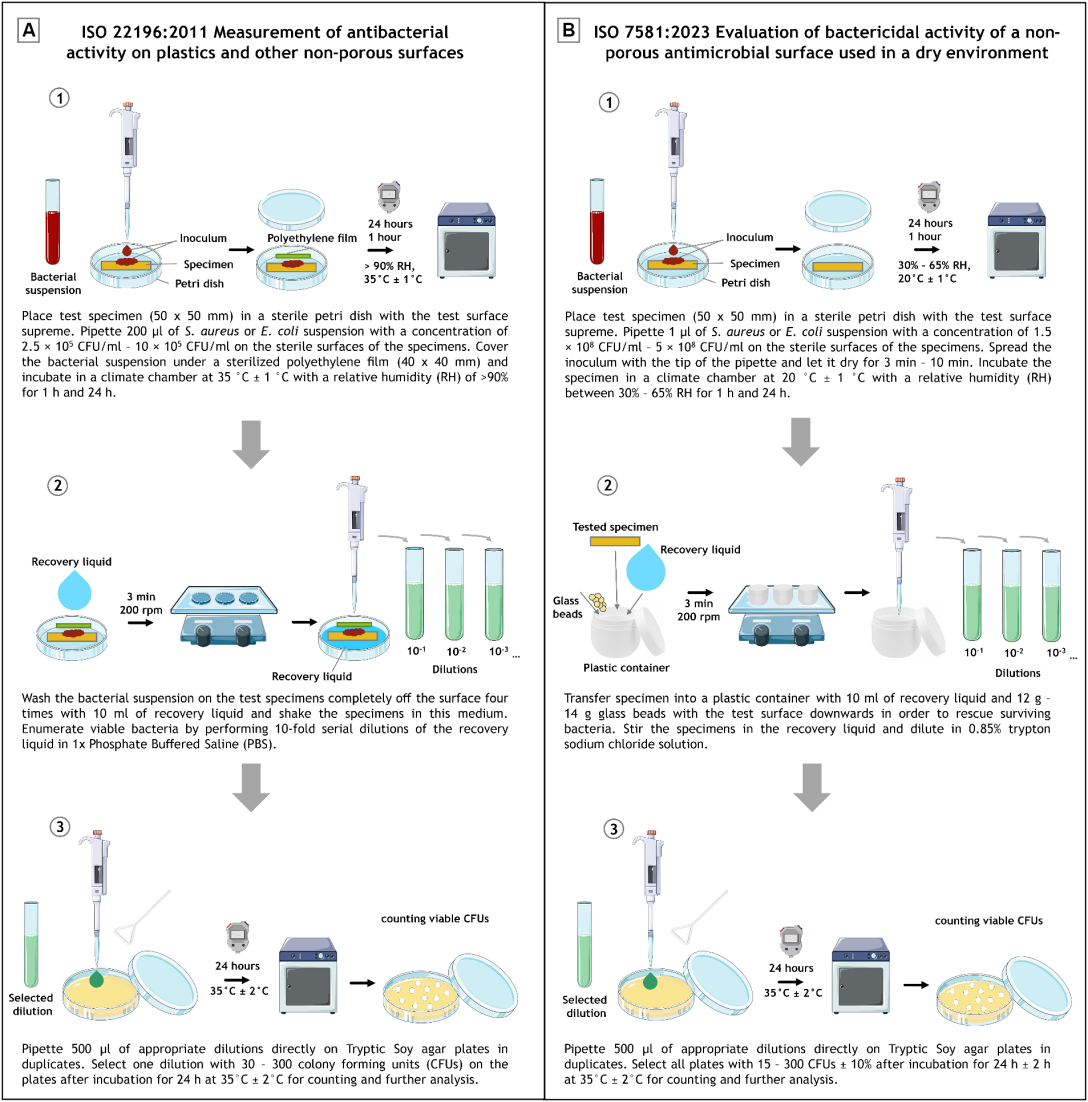
Schematic experimental set up depiction of ISO 22196:2011 **(A)** and ISO 7581:2023 **(B)**. Divided into (1) inoculation and incubation, (2) recovery and (3) plating and evaluation. The image was partly generated using Servier Medical Art, provided by Servier, licensed under a Creative Commons Attribution 3.0 unported license.

For a better comparison, the individual work steps of the two protocols were compared graphically (Figure 1).

Further, the most important work steps with commonalities and differences are listed in Table 1.

**Table 1:** Descripted are the main protocol details, common and different steps between ISO 22196:2011 and ISO 7581:2023.

	ISO 22196:2011	ISO 7581:2023
Bacterial strains	*S. aureus* DSM 346 (^*^DSM 799)	*S. aureus* DSM 799 (^*^DSM 346)
	*E. coli* DSM 1576 (^*^DSM 682)	*E. coli* DSM 682 (^*^DSM 1576)
	Pre-culture on Columbia blood agar plates	
Media	0.2% Tryptone Soy Broth (TSB, initial concentration)	0.85% Tryptone sodium chloride solution (Tryptone NaCl, initial concentration)
	1x Phosphate Buffered Saline (PBS, serial dilutions)	0.85% Tryptone sodium chloride solution (Tryptone NaCl, serial dilutions)
	Tryptic Soy Agar (TSA) plates	
Adjusting the initial bacterial suspension	*S. aureus*: 10^8^ CFU/mL	
	*E. coli*: 10^8^ CFU/mL	
Dilution of initial bacterial suspension	10^−4^ and 10^−5^ (serial dilution)	10^−6^ and 10^−7^ (serial dilution)
Specimens	In triplicates, tested uncoated and zinc-coated:	
	Low alloyed carbon steel	
	Polyethylene terephthalate (PET)	
	Glass	
Inoculation	Bacterial concentration: 2.5 × 10^5^–10 × 10^5^ CFU/mL	Bacterial concentration: 1.5 × 10^8^–5 × 10^8^ CFU/mL
	Volume: 200 μL	Volume: 1 μL
Test process	Cover and spread the bacterial suspension under a sterilized polyethylene film.	Spread the bacterial suspension with the tip of the pipette on the specimen surface.
		Let it dry for 3 min to max. 10 min.
		Start the incubation time points after drying process is completed.
Test conditions	35°C ± 1°C	20°C ± 1°C
	> 90% relative humidity (RH, climate chamber)	30–65% relative humidity (RH, climate chamber)
Time points	0 and 24 h (^*^1 h)	0 and 1 h (^*^24 h)
Recovery	Wash test specimens with 10 mL recovery liquid four times.	Transfer test specimens into a recipient with 10 mL recovery liquid and 12–14 g glass beads.
	Shake for 3 min with 200 rpm	
Serial dilutions	10^−1^–10^−3^ (serial dilution)	
Results in	CFU/cm^2^	CFU
Validation	-	Validation (V_n_): Pipette, instead of a bacterial suspension, 1 μL of Tryptone NaCl solution onto each specimen (for each strain, specimens and treatment), and 1 μL of test suspension is then added in plastic container.
Verification of the methodology	$\frac{\left(L_{\text{max}}-L_{\text{min}}\right)}{L_{\mathrm{mean}}}\leq 0.2$	$\frac{\left(C_{0h,\max}-C_{0h,\min}\right)}{\left(C_{0h,\mathrm{mean}}\right)}\leq 0.3$
Calculation of the antibacterial activity/log reduction	$R=U_{t}-A_{t}$	$R=C_{xh}-T_{xh}$
Evaluation	Select one dilution with 30–300 colony forming units (CFU)	Select all plates with 15–300 colony forming units (CFU) ± 10%

Information marked with (^*^) has been added for the respective ISO for better comparison between the two protocols.

#### 2.2.4 Further data analysis

Results were expressed as described in the ISOs (ISO 22196:2011 CFU/cm^2^; ISO 7581:2023 CFU) with corresponding mean ± standard deviation (SD). Depiction were generated using CorelDRAW 2019 (Corel Cooperation, Ottawa, Canada) or GraphPad Prism Version 10 (GraphPad Software, Boston, MA, United States).

## 3 Results

### 3.1 ISO 22196:2011 measurement of antibacterial activity on plastics and other non-porous surfaces

The low alloyed carbon steel, PET and glass samples were tested uncoated and coated with zinc to obtain the antibacterial activity of the zinc treated surfaces according to ISO 22196:2011.

**Figure 2:**
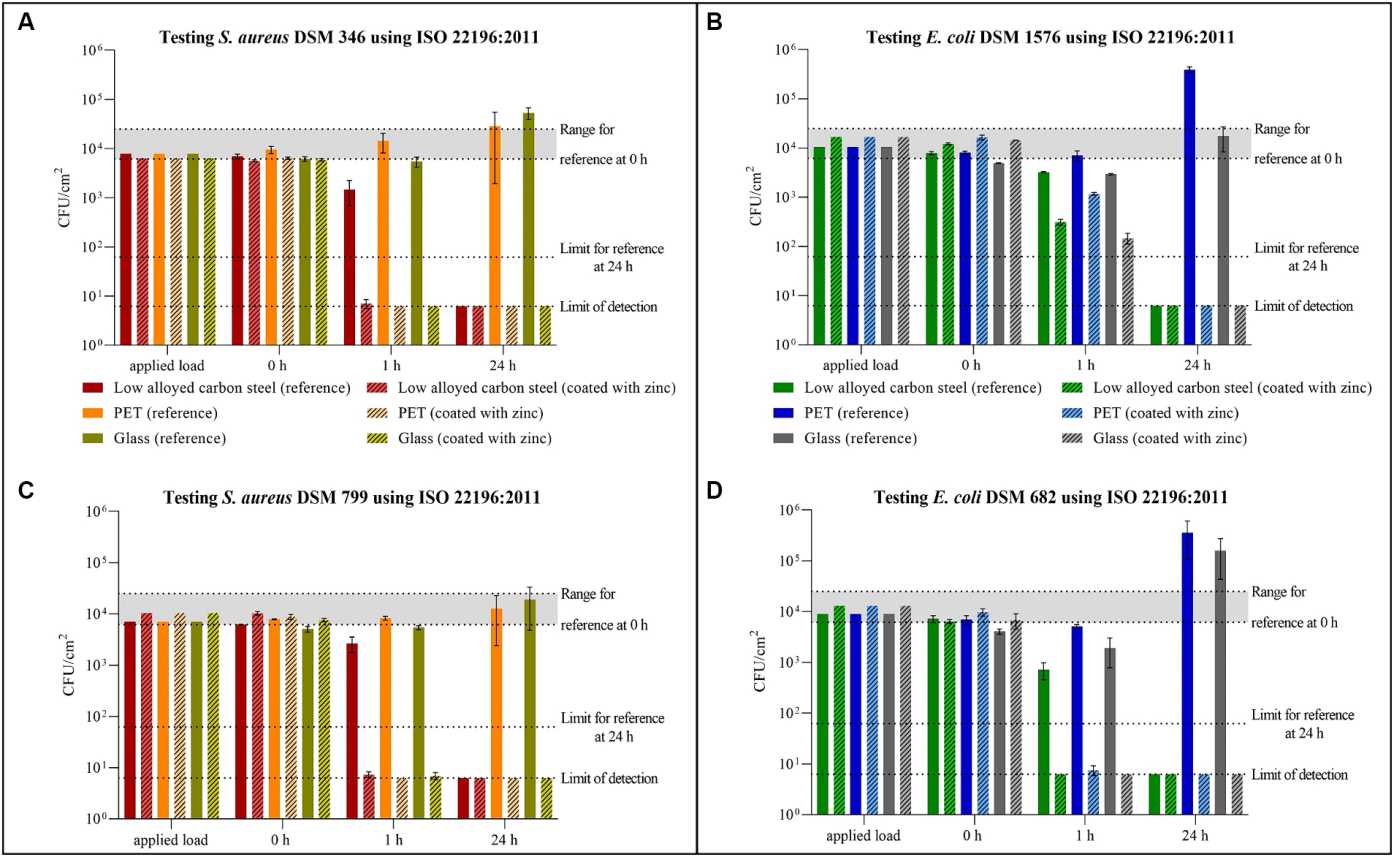
Quantification of *Staphylococcus aureus* DSM 346 **(A)** and DSM 799 **(C)**, and *Escherichia coli* DSM 1576 **(B)** and DSM 682 **(D)** after incubation on tested surfaces according to ISO 22196:2011. Specimens of low alloyed carbon steel, PET, and glass were compared uncoated (reference) and coated with zinc. The surfaces were incubated with a bacterial load of 2.5 × 10^5^–10 × 10^5^ CFU/mL (Applied load is given in CFU/cm^2^ for the applied volume of 200 μL). The temporal incubations (1 and 24 h) were performed at 35°C ± 1°C and RH of >90%. Further, 0 h shows the recovery immediately after inoculation on the tested specimens. After the time points, the bacteria were harvested and checked for their survival. The error bars indicate the standard errors of the respective means, which were composed of triplicates (*n* = 3). The limit of detection was set as 6.25 × 10^0^ CFU/cm^2^. Criteria for a valid test belonging to reference specimens are indicated with the range of viable bacteria that should be recovered at 0 h (6.2 × 10^3^ CFU/cm^2^ to 2.5 × 10^4^ CFU/cm^2^) and with the limit of detected bacteria after 24 h (6.2 × 10^1^ CFU/cm^2^).

All tested samples showed the same efficacy against both *S. aureus* strains. (Figures 2A,C). The uncoated surfaces (reference) of PET and glass did not show antibacterial efficacy. On the contrary, after 24 h a small increase of the bacterial concentration was evident. In contrast, no viable *S. aureus* was detectable on low alloyed carbon steel specimens after 24 h, regardless of the presence or absence of a zinc coating. However, a difference between the reference and the coated specimens of low alloyed carbon steel could be seen after the shorter incubation of 1 h. For the reference approximately 2 × 10^3^ CFU/cm^2^ were detectable, while the zinc-coated surfaces were below the detection limit of 6.25 × 10^0^ CFU/cm^2^. Similar to zinc-coated low alloyed carbon steel, the other coated surfaces also showed a good antibacterial effect against both *S. aureus* strains after 1 h incubation. The antibacterial activity/log reduction (Table 2) by comparing the uncoated with the coated surfaces of a substrate was thus between 1.5 × 10^3^ and 1.4 × 10^4^ CFU/cm^2^ for 1 h incubation period. *E. coli* DSM 682 (Figure 2D) behaved similarly to the *S. aureus* strains. No antibacterial effect was detectable on the reference surfaces of PET and glass. After 24 h neither on coated nor on uncoated low alloyed carbon steel surfaces bacteria of this *E. coli* strain were recognizable. The coated specimens showed a good antibacterial activity from 1 h incubation onwards. Even though the effect against *E. coli* DSM 682 and *E. coli* DSM 1576 did not differ after 24 h, different results were obtained between these two strains after an incubation time of 1 h on the coated surfaces. In addition, *E. coli* DSM 1576 still had at least 2,400 detectable colonies (= 1.5 × 10^2^ CFU/cm^2^) after 1 h on the zinc-coated surfaces (Figure 2B). The results of the antibacterial activity/log reduction for both *E. coli* strains are shown in Table 3.

**Table 2:** Antibacterial activity/log reduction calculated for both *Staphylococcus aureus* strains accordingly to ISO 22196:2011.

ISO 22196:2011	Low alloyed carbon steel		PET		Glass	
*S. aureus*	DSM 346	DSM 799	DSM 346	DSM 799	DSM 346	DSM 799
1 h	1.5 × 10^3^	2.6 × 10^3^	1.4 × 10^4^	8.2 × 10^3^	5.4 × 10^3^	5.4 × 10^3^
24 h	0	0	2.9 × 10^4^	1.3 × 10^4^	5.3 × 10^4^	1.9 × 10^4^

For results labeled “0,” the antibacterial activity/log reduction could not be calculated due to the existing effect on the corresponding reference sample.

**Table 3:** Antibacterial activity/log reduction calculated for both *Escherichia coli* strains accordingly to ISO 22196:2011.

ISO 22196:2011	Low alloyed carbon steel		PET		Glass	
*E. coli*	DSM 1576	DSM 682	DSM 1576	DSM 682	DSM 1576	DSM 682
1 h	2.9 × 10^3^	7.0 × 10^2^	5.9 × 10^3^	5.0 × 10^3^	2.8 × 10^3^	1.9 × 10^3^
24 h	0	0	3.9 × 10^5^	3.6 × 10^5^	1.8 × 10^4^	×10^5^

For results labeled “0,” the antibacterial activity/log reduction could not be calculated due to the existing effect on the corresponding reference sample.

The validity of all tests carried out in accordance with ISO 22196:2011 was confirmed by the 0 h triplicates (Supplementary Tables 1–4). In addition, the number of viable bacteria recovered at 0 h should be in the range of 6.2 × 10^3^ CFU/cm^2^ to 2.5 × 10^4^ CFU/cm^2^. In this study, three values were slightly below this range, but not below 4.1 × 10^3^ CFU/cm^2^ as shown in Supplementary Tables 1, 3. All three values belong to glass as reference, which indicated an application problem. The slight deviation of this validation criterion was therefore not taken into account. The third criterion states that the results of the reference samples should not be less than 6.2 × 10^1^ CFU/cm^2^ after 24 h. The low alloyed carbon steel reference samples had a better effect on all tested strains after 24 h, so the results were below this specification.

### 3.2 ISO 7581:2023 evaluation of bactericidal activity of a non-porous antimicrobial surface used in a dry environment

The same samples tested according to ISO 22196:2011 were also tested for their antibacterial activity under dry test conditions in accordance with ISO 7581:2023.

**Figure 3:**
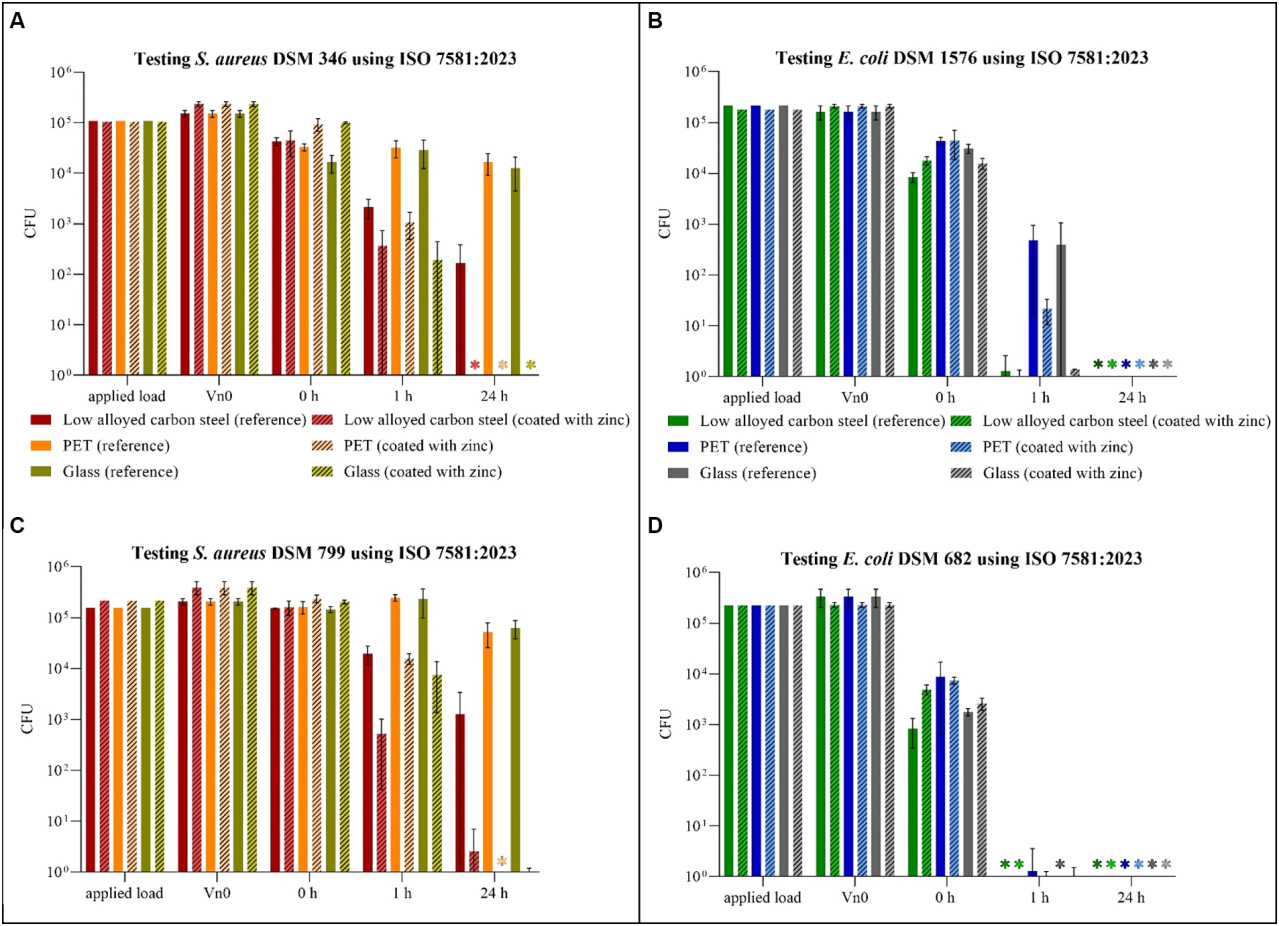
Quantification of *Staphylococcus aureus* DSM 346 **(A)** and DSM 799 **(C)** and *Escherichia coli* DSM 1576 **(B)** and DSM 682 **(D)** after incubation on tested surfaces according to ISO 7581:2023. Specimens of low alloyed carbon steel, PET and glass were compared uncoated (reference) and coated with zinc. The surfaces were incubated with a bacterial load of 1.5 × 10^8^–5 × 10^8^ CFU/mL (Applied load is given in CFU for the applied volume of 1 μL). The temporal incubations (1 and 24 h) were performed at 20°C ± 1°C and RH of 30–65%. Further, 0 h shows the recovery of bacteria immediately after drying on the tested specimens. After the time points, the bacteria were harvested and checked for their survival. The error bars indicate the standard errors of the respective means, which were composed of triplicates (*n* = 3). When no CFU were countable on each plate, including counts by filtration and counts from the surface embedded in the agar, the final result was set to 0 CFU and was marked with (^*^).

For *S aureus* DSM 346 (Figure 3A) and DSM 799 (Figure 3C), similar results were again obtained with this testing procedure. After 1 h under dry conditions, *S. aureus* remained relatively stable on the uncoated surfaces. Only on the low alloyed carbon steel reference a decrease of approximately 1 log_10_ was detectable compared to the initial concentration (0 h). A higher antibacterial efficacy for the same time point was recognizable on the coated specimens, whereas the difference between coated and uncoated surfaces became even clearer after 24 h. While colonies of *S. aureus* DSM 346 were not detectable on any of the coated surfaces, six colonies of *S. aureus* DSM 799 could still be detected on low alloyed carbon steel and one colony on glass using the filter method. Thus, a high antibacterial activity/log reduction (over 4 log_10_ CFU) as shown in Table 4 was found for PET and glass after 24 h. In contrast, low alloyed carbon steel only achieved a reduction of 1.7 × 10^2^ CFU (*S. aureus* DSM 346) and of 1.3 × 10^3^ CFU (*S. aureus* DSM 799) after 24 h, as the uncoated surfaces already had an effect. For the gram-negative bacteria (Figures 3B,D), no colonies could be detected on the agar plates after 24 h, neither on the uncoated nor on the zinc-coated surfaces. Therefore, the antibacterial activity/log reduction for 24 h was not calculable and was set to zero (Table 5). *Escherichia coli* DSM 682 also showed similar results for 1 h. Low alloyed carbon steel showed a complete reduction and no more bacteria were detectable. In the case of PET, however, three CFUs (uncoated) and one CFU (coated) were detectable on the filter. In the case of glass, only two CFUs were detectable on the coated surfaces, one by filtration and one by embedding the surface in agar. In contrast, *E. coli* DSM 1576 was able to survive longer on the tested surfaces. CFUs of this strain were detectable on all specimens after 1 h. The lowest decrease compared to the initial concentration (0 h) of bacteria concentration was observed on PET and glass reference samples. Therefore, an antibacterial activity/log reduction (4.6 × 10^2^ CFU for PET and 4.0 × 10^2^ CFU for glass) could only be calculated for these two samples for all results of the two *E. coli* strains (Table 5). In addition, both *E. coli* strains showed a reduction in countable CFU already after 0 h compared to *S. aureus*. For *E. coli* DSM 682 in particular, a reduction of 1.2 log_10_ to 2.2 log_10_ CFU was observed on the surfaces after this short drying time compared to the applied load. The survival of the gram-negative bacteria *E. coli* at the beginning of the experiment is therefore affected by the drying process and not by the effectiveness of the surfaces.

**Table 4:** Antibacterial activity/log reduction calculated for both *Staphylococcus aureus* strains accordingly to ISO 7581:2023.

ISO 7581:2023	Low alloyed carbon steel		PET		Glass	
*S. aureus*	DSM 346	DSM 799	DSM 346	DSM 799	DSM 346	DSM 799
1 h	1.8 × 10^3^	1.9 × 10^4^	3.1 × 10^4^	2.3 × 10^5^	2.9 × 10^4^	2.3 × 10^5^
24 h	1.7 × 10^2^	1.3 × 10^3^	1.7 × 10^4^	5.2 × 10^4^	1.3 × 10^4^	6.2 × 10^4^

**Table 5:** Antibacterial activity/log reduction calculated for both *Escherichia coli* strains accordingly to ISO 7581:2023.

ISO 7581:2023	Low alloyed carbon steel		PET		Glass	
*E. coli*	DSM 1576	DSM 682	DSM 1576	DSM 682	DSM 1576	DSM 682
1 h	0.8 × 10^0^	0	4.6 × 10^2^	0.8 × 10^0^	4.0 × 10^2^	−0.8 × 10^0^
24 h	0	0	0	0	0	0

For results labeled “0,” the antibacterial activity/log reduction could not be calculated due to the existing effect on the corresponding reference sample.

For the validity of the tests performed the initial suspension (X) as well as the number of theoretical CFUs deposited on the surface (N) should have a specified concentration range according to ISO 7581:2023. In this study, the concentrations for *S. aureus* DSM 346 were slightly below this prescribed range. However, as this was only a deviation of 0.4 log_10_, which occurred in both uncoated and coated specimens, it was not considered further (Supplementary Tables 5, 6). There was a further deviation in validity criterion number six. As this criterion could not be fulfilled due to the existing efficacy of the reference samples at certain incubation times and against certain bacterial strains (Supplementary Tables 5, 7), this deviation was also not taken into account. All other guidelines were met and the results can be considered valid.

## 4 Discussion

The application of antimicrobial surfaces requires the proof of their efficacy by *in vitro* methods in laboratories. The most common used protocol is ISO 22196:2011. However, as shown by other researchers, it has some significant limitations. Although the test procedure of the bacterial assay is quite easy to perform and does not require any specialized equipment, we would also like to point out shortcomings of the testing procedure that seemed particularly relevant to us. Moreover, we want to compare ISO 22196:2011 to ISO 7581:2023 and also list the advantages and disadvantages of this recently published standard:

### 4.1 Applying the bacteria to the test surface

The suitably cut polyethylene film facilitates the distribution of the bacterial suspension on the covered surfaces and enables the calculation of the results in CFU/cm^2^. However, it does not provide realistic test conditions, as the microorganisms would normally not be distributed evenly in a liquid phase over an inanimate surface area. Under practical/realistic conditions, the microorganisms would dry out on the surfaces, assumed that the products are not in continuous contact with water or moisture. Moreover, the wet exposure conditions with high surface area to inoculum volume ratio are known to overestimate antimicrobial activity compared to dry exposure (Kaur et al., 2023). As described by Bäumler et al. (2022), the wet testing method should be replaced/enhanced by testing under “more realistic” and dry conditions. The newly published ISO 7581:2023 offers exactly that—antibacterial testing in a dry environment. However, having carried out tests in our laboratory in accordance with this standard, we believe that this test method could also be improved. Applying such a small volume (1 μL bacterial suspension) as recommended by ISO 7581:2023 turned out to be very difficult. Firstly, volumes below 20 μL can lead to errors (Guan et al., 2023). In our case, a 0.5–10 μL Eppendorf Research plus pipette was used for applying 1 μL on the tested surfaces, which is specified by the manufactures to have a systematic error of ±0.12 μL. Due to the small volume with high bacterial concentration, this error has a significant effect on the actual applied bacterial load. Moreover, in case of ISO 7581:2023 the small volume should be additionally distributed on a dry surface with the pipette tip, leaving residues of the bacterial suspension in and on the tip. A complete distribution of the test-volume to the surfaces is therefore often not possible and the reproducibility of the inoculum quantity and thus also the number of the applied CFUs cannot be guaranteed (Cunliffe et al., 2021). Secondly, the distribution and drying process strongly depends on the respective surface material. As summarized by Bäumler et al. (2022) the drying can take seconds but also up to minutes. During our tests, there was also a drying range of <1–9 min depending on the material used and the possibility of distribution on the surface. For instance we could see, that uncoated surfaces needed more time for the drying process than the zinc-coated specimens, probably caused by inadequate distribution through the uncoated and more hydrophobic surfaces. In addition, differences between the triplicates of one data point were also present. With the same application technique, the drying time varied up to a maximum of 2 min between the triplicates of a test series. Finally, it was not recognizable when the bacterial suspension had dried out, particularly in case of PET coated with zinc due to the irregular particle loading (only 36% of the surfaces were covered with zinc particles). As a result, high deviations were already recorded after 0 h for this sample type, as some surfaces were still moist when they were placed in the recovery liquid. These differences could be caused by two mechanisms: Firstly, bacteria such as *E. coli* die quickly under dry conditions (Hirai, 1991), and secondly, prolonged humid conditions can increase the release of biocidal substances from the coating by diffusion (Bäumler et al., 2022). All the points described above summarized, applying the bacterial suspension only according to ISO 22196:2011 or according to ISO 7581:2023 seems not suitable enough for us to test non-porous AMMs. One possibility for improvement would be to apply higher volumes on the surfaces. Some researchers have already published modified testing protocols from the existing ISO 22196:2011, using the large-droplet inoculation (LDI) method (Caschera and Lukasz, 2016; Caschera et al., 2021; Kaur et al., 2023) or a touch transfer assay (Knobloch et al., 2017). For the LDI method, 50 or 100 μL droplets are applied on the surfaces. However, this increases the drying time and thus the incubation time to 3 or 24 h until the surfaces are complete dry again, which in turn can increase the antimicrobial efficacy. In our opinion, the use of an aerosol chamber would allow a uniform and reproducible application method with a fast drying time (Ronan et al., 2013). As already show in our laboratory, spraying bacterial suspensions with a simple manual pump spray or with an automatic TLC sampler allows a homogenous distribution of the bacteria on the test plate (Windisch et al., 2024).

### 4.2 Incubation period

Another point we criticize about the protocol is the long incubation time of 24 h specified in ISO 22196:2011. This long incubation time often results in a significant reduction in the bacterial count of the uncoated reference samples. In our study, for example, the uncoated low alloyed carbon steel samples showed a complete reduction in *S. aureus* and *E. coli* for ISO 22196:2011 after 24 h. From this point of view, the uncoated samples would have the same effectiveness as the zinc coated low alloyed carbon steel surfaces. They would therefore be ruled out as a reference, (according to ISO 22196:2011 for reference samples not less than 6.2 × 10^1^ CFU/cm^2^ should be detected after 24 h) and the effectiveness of a coating would not be determined at all—except with shorter test period (1 h). As recently described by Behzadinasab et al. (2023), an effective antimicrobial coating must eliminate microorganisms within minutes or even faster, depending on how frequently users touch the coated object. This is particularly important for materials used in healthcare settings were surfaces close to patient environment that are frequently touched (“high-touch surfaces”) allow transmission from animated sources to other via contaminated inanimate surfaces (Kramer and Assadian, 2014). In our view, the testing of antimicrobial surfaces with short time points should be an essential part of all testing protocols. Only with short time points of e.g., 0.5, 1, and 3 h can an efficacy kinetics be derived.

In addition to the different application strategies and incubation time points, the two ISOs are generally difficult to compare. For instance, the nutrient concentration of the application media used on the surfaces varies with 0.2% for ISO 22196:2011 and 0.85% for ISO 7581:2023. The higher nutrient content could lead to growth over longer incubation periods and influence the results (Wiegand et al., 2018). A very interesting difference between the two standards is the use of different test strains. In our investigations, the *E. coli* strains in particular showed differences in several cases. *E. coli* DSM 1576 (ISO 22196:2011) is more stable and survives longer than *E. coli* DSM 682 (ISO 7581:2023; Figures 2, 3). In general, gram-negative bacteria cannot survive as long as gram-positive bacteria under dry conditions. For instance, Hirai (1991) described that the number of *E. coli* rapidly decreased and after 7 h no viable cells could be found on glass plates, which is also consistent with our results. Thus, microorganisms such as *S. aureus*, which can persist longer at low humidity (McDade and Hall, 1964) should be preferred for testing according to ISO 7581:2023. In addition, when testing disinfectants, a higher test organism spectrum is specified, which also includes other microorganisms such as *Candida albicans*, *Mycobacterium* spp., and *Pseudomonas aeruginosa*. Testing more microorganisms according to “Deutsche Gesellschaft für Hygiene und Mikrobiologie e.V.” would also improve the statement of the antimicrobial efficacy of non-porous surfaces. Furthermore, small adjustments to the protocols, as carried out in our laboratory before, enable tests against bacteriophages which can be used as model organisms for human viruses and can prove the antiviral efficacy of the surfaces (Poelzl et al., 2023).

### 4.3 Evaluation and validation

Further differences can be found in the evaluation. Firstly, the number of colonies that are allowed to be counted on the agar plates varies with 30–300 CFU (ISO 22196:2011) to 14–330 CFU (ISO 7581:2023). The general accepted ranges are 30–300 CFU or 25–250 CFU per plate (Sutton, 2012) and are therefore in accordance with ISO 22196:2011. Secondly, the results are stated either in CFU/cm^2^ for the wet testing conditions under the 40 mm × 40 mm polyethylene film (ISO 22196:2011) or in CFU for the dry testing conditions of ISO 7581:2023. Standardization would be desirable in both cases.

A further evaluation strategy for ISO 7581:2023 yields a further deviation. While ISO 22196:2011 sets a limit of detection with the used volume of the recovery liquid (number of colonies should be counted as “< V”), ISO 7581:2023 requires the remaining recovery liquid to be filtered and the tested surface to be embedded in agar. By analyzing the entire medium and the used surface itself, the limit of detection can be avoided and a complete reduction can actually be determined, if no CFUs were countable on any of the plates. In our tests, individual bacteria were detected using the very laborious filtration method. However, overgrown filters made an evaluation often impossible (Supplementary Figure 2). The additional evaluations in which the samples have to be enclosed in agar were hardly feasible, as the colonies were difficult to count (e.g., blurred colonies, dark background, or changes of the coating due to oxidation, Supplementary Figure 3) and therefore could not be included to the evaluation and calculation. In general, the evaluation without filtration and extra procedures is very accurate with a selected detection limit.

There are also major differences between the two standards in terms of validation. One common feature is the specification of the range the bacterial suspension should initially have and the calculation of the validity of the test using the 0 h triplicates to eliminate inaccuracies. All other criteria differ between ISO 22196:2011 and ISO 7581:2023. Some of them are occasionally incomprehensible and difficult to fulfill. For instance, the references pose a major difficulty. Although they have no active ingredient, they show an antimicrobial effect over longer incubation periods (often only due to the physiological characteristics of the test strains) and therefore become a knock-out criterion for testing. Moreover, the calculation of the antibacterial activity/log reduction is also not possible with these samples due to the calculation specification of both ISOs. We would recommend to calculate the antibacterial activity/log reduction by the initial concentration (0 h) for each sample type to generate results for reference and coated samples separatly. In addition, the use of an additionally reference sample would also be appropriate here, which has already been repeatedly proven to have no antimicrobial effect and thus can be used as negative control, e.g., glass (Kaur et al., 2023).

To conclude, ISO 22196:2011 and ISO 7581:2023 represent two completely different approaches to test non-porous surfaces for their antimicrobial efficacy. Neither of these two test protocols can be described as better or more application-oriented, since both have limitations. It would be better to adapt the test conditions to the subsequent application in order to select a suitable test method. Adaptations to the ISOs should be made, including incubation conditions (time, temperature, or relative humidity), testing strains and volume, to generate appropriate and more realistic conditions. If the test conditions are not optimized for the test product, the effectiveness of the product will be over-or underestimated.

## Data availability statement

The original contributions presented in the study are included in the article/Supplementary material, further inquiries can be directed to the corresponding author.

## Author contributions

SM: Conceptualization, Writing – original draft, Writing – review & editing. SP: Conceptualization, Data curation, Formal analysis, Methodology, Writing – original draft, Writing – review & editing. DD: Data curation, Formal analysis, Methodology, Writing – review & editing. EZ: Data curation, Formal analysis, Methodology, Writing – review & editing. CK: Funding acquisition, Project administration, Supervision, Writing – review & editing.
