# Supplementary material for: Antimicrobial non-porous surfaces: a comparison of the standards ISO 22196:2011 and the recently published ISO 7581:2023
Source: Front Microbiol. 2024 Jul 17;15:1400265. doi: 10.3389/fmicb.2024.1400265 (PMC11288859; doi:10.3389/fmicb.2024.1400265)
Supplement: Supplementary file 1 [file Data_Sheet_1.PDF]

Supplementary Material

1.1 Depiction of samples

All sample types used within this study are shown in Supplementary Figure 1.

**Low alloyed carbon steel**	**PET**	**Glass**
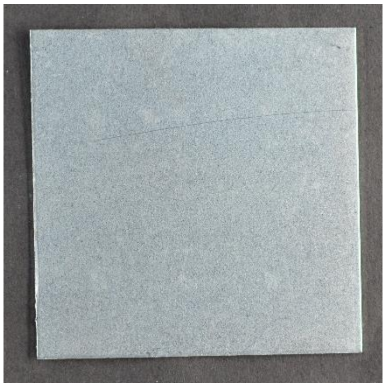	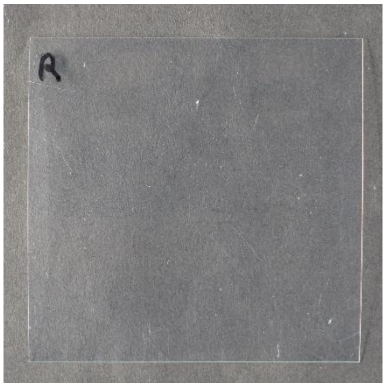	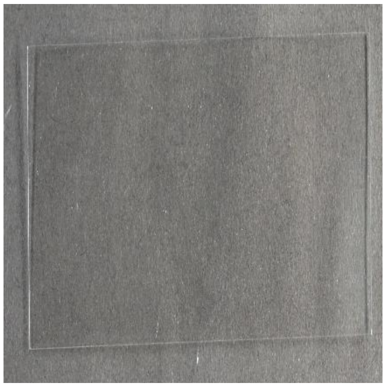
Reference (uncoated)	Reference (uncoated)	Reference (uncoated)
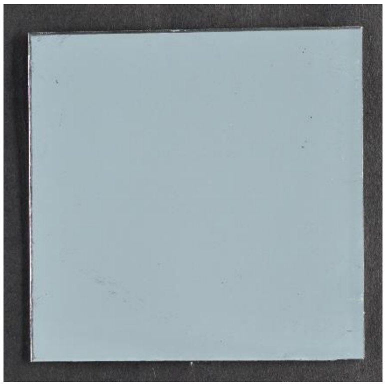	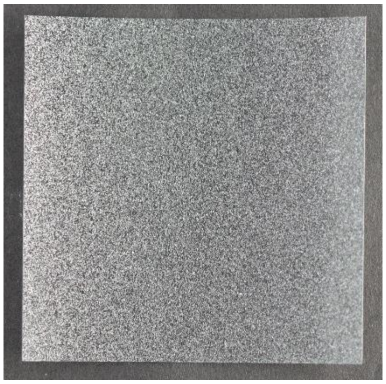	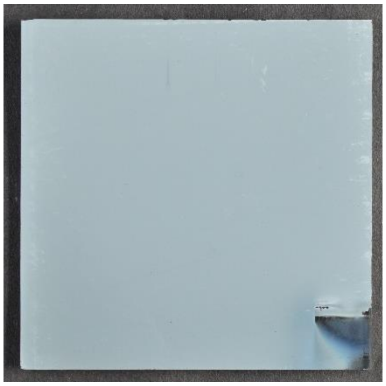
Coated with 2 µm zinc	Coated with 36% zinc particle loading	Coated with 2 µm zinc

Supplementary Figure 1 Depiction of uncoated (reference) and coated specimens of low alloyed carbon steel, PET and glass

1.2 Additional evaluation strategy according to ISO 7581:2023

Filters with colonies of *S. aureus* or *E. coli* after filtering the remaining recovery liquid according to ISO 7581:2023 are shown in Supplementary Figure 2.

S. aureus		***E. coli***	
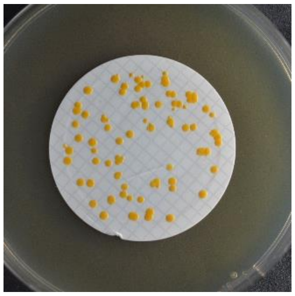	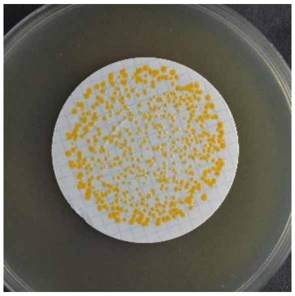	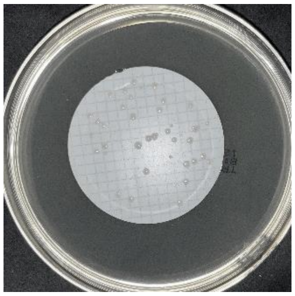	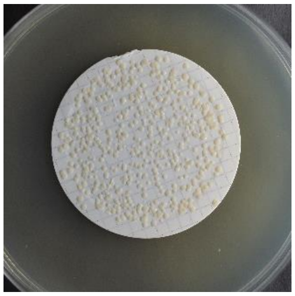

Supplementary Figure 2 Exemplary results from the filter method with few and many colonies of both tested bacteria species

Tested samples enclosed in agar to be able to count still viable colonies on the specimen’s surface (ISO 7581:2023) are exemplary shown in Supplementary Figure 3.

**Low alloyed carbon steel**	**PET**	**Glass**
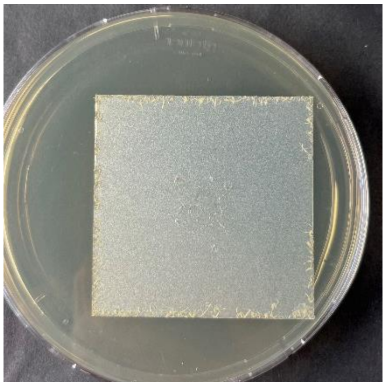	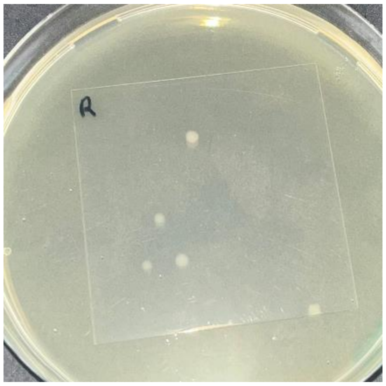	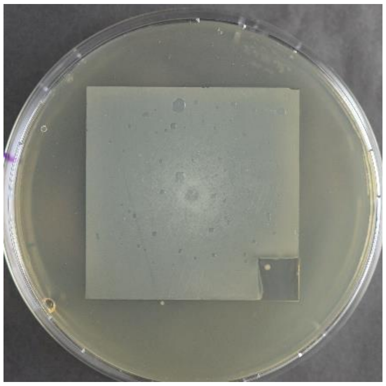
Uncoated without colonies (inoculation site)	Uncoated with five *E. coli* colonies	Coated with two *S. aureus* colonies (inoculation site)
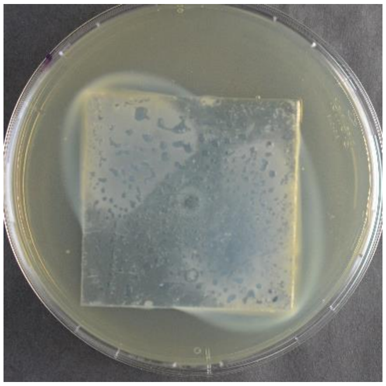	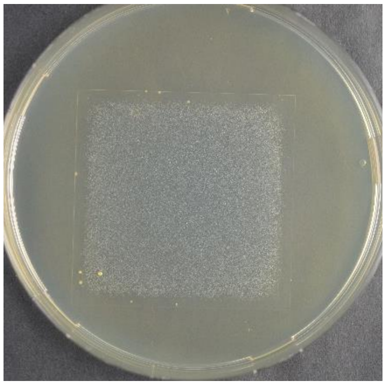	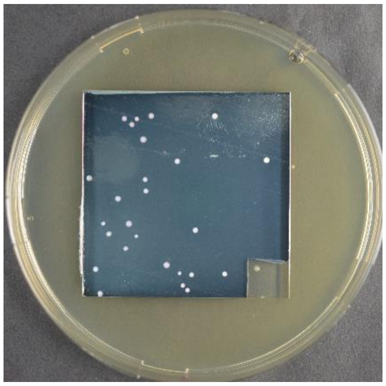

Changes of the coating in agar (colonies not recognizable)	Coated with ten *S. aureus* colonies	Coated with 29 *S. aureus* colonies (backside)

Supplementary Figure 3 Exemplary images of the specimens embedded in agar showing well countable colonies to altered surfaces making counting impossible

1.3 Validation criteria according to ISO 22196:2011

1. The verification of the methodology was calculated through the 0 h triplicates with the following formula:

$$\frac{(L_{max} - L_{min})}{L_{mean}} \leq 0.2$$

L_{max} 10 logarithm of the maximum number of viable bacteria found on a specimen

L_{min} 10 logarithm of the minimum number of viable bacteria found on a specimen

L_{mean} 10 logarithm of the mean number of viable bacteria found on the specimens

A value ≤ 0.2 indicated a valid test result.

2. The number of viable bacteria recovered at time point 0 h should be within the range of 6.2×10^3 CFU/cm^2 to 2.5×10^4 CFU/cm^2 (only for reference specimens).

3. After 24 h the results of the reference specimens should not be less than 6.2×10^1 CFU/cm^2.

Supplementary Table 1 Results for the validation criteria from *S. aureus* DSM 346 & DSM 799 on reference samples according to ISO 22196:2011. Data marked in red do not fulfil the conditions for a valid test.

**ISO 22196:2011** ***S. aureus***	**Low alloyed carbon steel**		**PET**		**Glass**	
	DSM 346	DSM 799	DSM 346	DSM 799	DSM 346	DSM 799
1.	0.02	0.00	0.03	0.00	0.02	0.03
2.	7.0×10^3 CFU/cm^2	6.2×10^3 CFU/cm^2	9.5×10^3 CFU/cm^2	7.9×10^3 CFU/cm^2	6.2×10^3 CFU/cm^2	5.1×10^3 CFU/cm^2
3.	6.3×10^0 CFU/cm^2	6.3×10^0 CFU/cm^2	2.9×10^4 CFU/cm^2	1.3×10^4 CFU/cm^2	5.3×10^4 CFU/cm^2	1.9×10^4 CFU/cm^2

Supplementary Table 2 Results for the validation criteria from *S. aureus* DSM 346 & DSM 799 on coated samples according to ISO 22196:2011. The second and third criteria are only specified for reference specimens.

**ISO 22196:2011** ***S. aureus***	**Low alloyed carbon steel + zinc**		**PET + zinc**		**Glass + zinc**	
	DSM 346	DSM 799	DSM 346	DSM 799	DSM 346	DSM 799
1.	0.01	0.02	0.01	0.02	0.01	0.02
2.	-	-	-	-	-	-
3.	-	-	-	-	-	-

Supplementary Table 3 Results for the validation criteria from *E. coli* DSM 1576 & DSM 682 on reference samples according to ISO 22196:2011. Data marked in red do not fulfil the conditions for a valid test.

**ISO 22196:2011** ***E. coli***	**Low alloyed carbon steel**		**PET**		**Glass**	
	DSM 1576	DSM 682	DSM 1576	DSM 682	DSM 1576	DSM 682
1.	0.02	0.03	0.01	0.04	0.01	0.03
2.	7.9×10^3 CFU/cm^2	7.2×10^3 CFU/cm^2	8.1×10^3 CFU/cm^2	7.0×10^3 CFU/cm^2	4.9×10^3 CFU/cm^2	4.1×10^3 CFU/cm^2
3.	6.3×10^0 CFU/cm^2	6.3×10^0 CFU/cm^2	3.9×10^5 CFU/cm^2	3.6×10^5 CFU/cm^2	1.8×10^4 CFU/cm^2	1.6×10^5 CFU/cm^2

Supplementary Table 4 Results for the validation criteria from *E. coli* DSM 1576 & DSM 682 on coated samples according to ISO 22196:2011. The second and third criteria are only specified for reference specimens.

**ISO 22196:2011 *E. coli***	**Low alloyed carbon steel + zinc**		**PET + zinc**		**Glass + zinc**	
	DSM 1576	DSM 682	DSM 1576	DSM 682	DSM 1576	DSM 682
1.	0.01	0.02	0.02	0.03	0.00	0.07
2.	-	-	-	-	-	-
3.	-	-	-	-	-	-

1.4 Validation criteria according to ISO 7581:2023

1. X (the initial suspension) shall be between 1.5×10^8 and 5×10^8 CFU.

2. N (number of theoretical CFUs deposited on the surface) shall be between 1.5×10^5 and 5×10^5 CFU.

3. V_n shall differ from N by < 2 log

4. The difference between the V_n surfaces shall not be > 0.3 log.

5. C_0 triplicates were used to verify the methodology through the following formula:

$$\frac{(C_{0h,max} - C_{0h,min})}{(C_{0h,mean})} \leq 0.3$$

$C_{0h,\,max}$ 10 logarithm of the maximum number of viable bacteria found on a specimen

$C_{0h,\,min}$ 10 logarithm of the minimum number of viable bacteria found on a specimen

$C_{0h,\,mean}$ 10 logarithm of the mean number of viable bacteria found on the specimens

A value ≤ 0.3 indicated a valid test result.

6. The value of the mean log – SD of C_{0h} and C_{xh} (only reference specimens) shall be ≥ 3.

7. S (surfaces enclosed in agar) shall be less than 100 CFU for active surfaces.

8. Check of the counts obtained by the weighted mean: the quotient is neither less than 5 nor more than 15 (incomprehensible description, marked with ‘‘?’’).

Supplementary Table 5 Results for the validation criteria from *S. aureus* DSM 346 & DSM 799 on reference samples according to ISO 7581:2023. Data marked in red do not fulfil the conditions for a valid test. The seventh criterion is only specified for coated specimens. The eighth criterion is described incomprehensibly and was therefore marked with ‘‘?’’.

ISO 7581:2023 *S. aureus*	Low alloyed carbon steel		PET		Glass	
	DSM 346	DSM 799	DSM 346	DSM 799	DSM 346	DSM 799
1.	1.1×10^8 CFU	1.6×10^8 CFU	1.1×10^8 CFU	1.6×10^8 CFU	1.1×10^8 CFU	1.6×10^8 CFU
2.	1.1×10^5 CFU	1.6×10^5 CFU	1.1×10^5 CFU	1.6×10^5 CFU	1.1×10^5 CFU	1.6×10^5 CFU
3.	0.7×10^0	0.8×10^0	0.7×10^0	0.8×10^0	0.7×10^0	0.8×10^0
4.	1.4×10^0	1.3×10^0	1.4×10^0	1.3×10^0	1.4×10^0	1.3×10^0
5.	0.03	0.00	0.03	0.04	0.03	0.04
6.	0 h: 3.6×10^4<br>1 h: 1.4×10^3<br>24 h: -6.5×10^0	0 h: 1.5×10^5<br>1 h: 1.4×10^4<br>24 h: -4.3×10^2	0 h: 2.9×10^4<br>1 h: 2.2×10^4<br>24 h: 1.1×10^4	0 h: 1.3×10^5<br>1 h: 2.2×10^5<br>24 h: 3.1×10^4	0 h: 1.1×10^4<br>1 h: 1.6×10^4<br>24 h: 6.0×10^3	0 h: 1.3×10^5<br>1 h: 1.2×10^5<br>24 h: 3.8×10^4
7.	-	-	-	-	-	-
8.	?	?	?	?	?	?

Supplementary Table 6 Results for the validation criteria from *S. aureus* DSM 346 & DSM 799 on coated samples according to ISO 7581:2023. Data marked in red do not fulfil the conditions

for a valid test. The fifth and sixth criteria are only specified for reference specimens. The eighth criterion is described incomprehensibly and was therefore marked with ‘‘?’’.

**ISO 7581:2023** ***S. aureus***	**Low alloyed carbon steel + zinc**		**PET + zinc**		**Glass + zinc**	
	DSM 346	DSM 799	DSM 346	DSM 799	DSM 346	DSM 799
1.	1.1×10^8 CFU	2.2×10^8 CFU	1.1×10^8 CFU	2.2×10^8 CFU	1.1×10^8 CFU	2.2×10^8 CFU
2.	1.1×10^5 CFU	1.6×10^5 CFU	1.1×10^5 CFU	1.6×10^5 CFU	1.1×10^5 CFU	1.6×10^5 CFU
3.	0.5×10^0	0.6×10^0	0.5×10^0	0.6×10^0	0.5×10^0	0.6×10^0
4.	1.2×10^0	1.8×10^0	1.2×10^0	1.8×10^0	1.2×10^0	1.8×10^0
5.	-	-	-	-	-	-
6.	-	-	-	-	-	-
7.	< 100 CFU	< 100 CFU	< 100 CFU	< 100 CFU	< 100 CFU	< 100 CFU
8.	?	?	?	?	?	?

Supplementary Table 7 Results for the validation criteria from *E. coli* DSM 1576 & DSM 682 on reference samples according to ISO 7581:2023. Data marked in red do not fulfil the conditions for a valid test. The seventh criterion is only specified for coated specimens. The eighth criterion is described incomprehensibly and was therefore marked with ‘‘?’’.

**ISO 7581:2023** ***E. coli***	**Low alloyed carbon steel**		**PET**		**Glass**	
	DSM 1576	DSM 682	DSM 1576	DSM 682	DSM 1576	DSM 682
1.	2.2×10^8 CFU	2.2×10^8 CFU	2.2×10^8 CFU	2.2×10^8 CFU	2.2×10^8 CFU	2.2×10^8 CFU

2.	2.2×10^5 CFU	2.2×10^5 CFU	2.2×10^5 CFU	2.2×10^5 CFU	2.2×10^5 CFU	2.2×10^5 CFU
3.	1.7×10^0	0.7×10^0	1.7×10^0	0.7×10^0	1.7×10^0	0.7×10^0
4.	1.9×10^0	2.2×10^0	1.9×10^0	2.2×10^0	1.9×10^0	2.2×10^0
5.	0.02	0.19	0.03	0.03	0.04	0.05
6.	0 h: 6.9×10^3<br>1 h: 0.2×10^0<br>24 h: 0.0×10^0	0 h: 4.3×10^2<br>1 h: 0.0×10^0<br>24 h: 0.0×10^0	0 h: 3.8×10^4<br>1 h: 1.0×10^2<br>24 h: 0.0×10^0	0 h: 2.1×10^3<br>1 h: 0.5×10^0<br>24 h: 0.0×10^0	0 h: 2.6×10^4<br>1 h: -1.4×10^2<br>24 h: 0.0×10^0	0 h: 1.5×10^3<br>1 h: 0.0×10^0<br>24 h: 0.0×10^0
7.	-	-	-	-	-	-
8.	?	?	?	?	?	?

Supplementary Table 8 Results for the validation criteria from *E. coli* DSM 1576 & DSM 682 on coated samples according to ISO 7581:2023. Data marked in red do not fulfil the conditions for a valid test. The fifth and sixth criteria are only specified for reference specimens. The eighth criterion is described incomprehensibly and was therefore marked with ‘‘?’’.

**ISO 7581:2023** ***E. coli***	**Low alloyed carbon steel + zinc**		**PET + zinc**		**Glass + zinc**	
	DSM 1576	DSM 682	DSM 1576	DSM 682	DSM 1576	DSM 682
1.	1.8×10^8 CFU	2.3×10^8 CFU	1.8×10^8 CFU	2.3×10^8 CFU	1.8×10^8 CFU	2.3×10^8 CFU
2.	1.8×10^5 CFU	2.3×10^5 CFU	1.8×10^5 CFU	2.3×10^5 CFU	1.8×10^5 CFU	2.3×10^5 CFU
3.	0.9×10^0	0.6×10^0	0.9×10^0	0.6×10^0	0.9×10^0	0.6×10^0
4.	1.2×10^0	1.1×10^0	1.2×10^0	1.1×10^0	1.2×10^0	1.1×10^0

5.	-	-	-	-	-	-
6.	-	-	-	-	-	-
7.	< 100 CFU	< 100 CFU	< 100 CFU	< 100 CFU	< 100 CFU	< 100 CFU
8.	?	?	?	?	?	?
